# Exclusive breastfeeding: Facilitators and barriers from the maternal perspective

**DOI:** 10.18332/ejm/222630

**Published:** 2026-07-21

**Authors:** Ianina Tuñón, Nicolas Zabalza Sammarone, Aldana Boragnio

**Affiliations:** 1 Observatory of the Argentine Social Debt, Pontifical Catholic University of Argentina, Buenos Aires, Argentina

**Keywords:** exclusive breastfeeding, breastfeeding barriers, maternal health, infant development, socioeconomic context

## Abstract

**INTRODUCTION:**

Exclusive breastfeeding (EBF) is a cornerstone for infant development and maternal health, offering nutritional, emotional, and public health benefits. Despite its importance, global EBF rates remain below targets, with barriers varying by socioeconomic context. In Argentina, while 91.7% of infants start breastfeeding, only 44.6% sustain EBF at six months.

**METHODS:**

A qualitative study was conducted using 32 focus groups involving 231 mothers from four Argentine urban areas with varying EBF prevalence. Participants were segmented by maternal experience, education level, and breastfeeding continuity. Thematic analysis was performed using MAXQDA and Atlas.ti, identifying key facilitators and barriers to EBF.

**RESULTS:**

Key facilitators included prenatal breastfeeding education, postpartum support, conducive workplace policies, and strengthened mother–infant bonding. Barriers encompassed physical pain, psychological distress, delivery complications, workplace tensions, and sociocultural pressures such as the perception of insufficient milk supply. Digital platforms emerged as vital tools for information exchange and support, particularly for primipara mothers, although insufficient public policies limit their impact on sustaining EBF.

**CONCLUSIONS:**

Access to education, professional guidance, and digital support platforms significantly contributes to EBF success. However, persistent challenges, including family dynamics, cultural stigmas, and work-related tensions, undermine EBF continuity. Comprehensive public policies promoting shared responsibility among families, employers, and society are critical to sustaining EBF practices. Targeted interventions addressing these multifaceted challenges can enhance maternal and child health outcomes globally.

## INTRODUCTION

Breastfeeding (BF) is a feeding method human beings initiate when coming to the world, but it is also a public health strategy which prioritizes infant development and maternal health throughout both mother and infant’s lives^1^. There are multiple benefits from BF, including immunity, nutrition, emotional and psychological well-being and public health^2^.

Recent studies have emphasized that the decision to initiate and sustain exclusive breastfeeding is strongly influenced by structural, social, and interpersonal factors, beyond mere intention^1-3^. Elements such as work-related constraints, previous breastfeeding difficulties, emotional vulnerability during the postpartum period, and the presence or absence of social support – particularly from close family members – have been shown to play a central role^4-6^. These findings support the need for in-depth, contextualized analyses that highlight the lived experiences of mothers.

Over the last decade, the prevalence of exclusive breastfeeding (EBF) has increased. It is estimated that it has reached 48% of infants and young children^7^. Even though many countries – rich and poor alike – have not made much progress towards reaching the global target of 70%, in high-income countries, the prevalence of mothers who have ever breastfed is much lower than in low-income countries. BF is one of the few positive health behaviors whose prevalence in poorer countries is higher than in richer ones; also, poor women breastfeed for longer than wealthier women in low- and middle-income countries. On the contrary, infant feeding modalities with breast milk substitutes act oppositely in richer countries, with rates being higher among wealthier and more educated women^8,9^.

Low EBF rates are of concern to states. In poorer countries, the main challenge is delayed breastfeeding initiation; just one in two neonates is put to the breast within the first hour of birth. In contrast, in middle- and high-income countries, where less than one in five infants is breastfed for the first 12 months, the challenges are different. Simultaneously, the data surrounding BF are limited, which hinders trend and progress monitoring^10^.

In the case of Argentina, these issues take place differently. Argentina is one of the main Latin American economies. The World Bank’s income classification sets Argentina as a middle-high-income country, even though its productivity matrix is centered on activities of low and medium-low technological complexity, in a productive structure that presents high levels of concentration and foreign capital^11^. Also, poverty and indigence rates affect 66% and 27% of children aged 0–14 years, respectively^12^. According to the National Survey on Breastfeeding (EnaLac)^13^, breastfeeding trends have remained stable since 2017. With 91.7% of infants initiating their feeding with breast milk, it is possible to say that, in Argentina, there is a high practice of BF. Nevertheless, EBF decreases as the age of infants increases: 53.5% of 2-month-old infants have EBF, but this number decreases to 44.6% when they are 6-month-old babies^13^. Moreover, data show that as EBF decreases – namely, from the second month – mixed feeding (alternating human milk with other milk) increases. This clearly shows that the initiation of breastfeeding is not the central problem, but rather its maintenance is what entails a significant challenge that needs to be focused on.

Argentina holds a wide range of legislation promoting BF. The National Constitution (in its Article 75, subsection 23) protects women who breastfeed, while also Act No. 26061, in its Article 18, extends the comprehensive protection measures to ‘the mother and the father during pregnancy, labor and the lactation period’. Act No. 27611 on ‘Comprehensive Health Care and Assistance during Pregnancy and Early Childhood’ aims to ‘protect, strengthen and support comprehensive care to protect the life and health of pregnant persons and children up to 3 years of age’^14^. Moreover, labor legislation establishes the right to have breastfeeding breaks (Law No. 20744, Article 179) and suitable spaces for the extraction and conservation of human milk (Law No. 26873 ‘Breastfeeding Promotion and Public Awareness’).

As it is known, breastfeeding is conditioned by multiple barriers (including physical and emotional challenges, among others) during its initiation and maintenance. With regard to the antecedents, there is diverse research on the actions conducted worldwide in order for women to choose and maintain breastfeeding^1^, as well as on the benefits and difficulties that arise in BF based on economic and socio-individual nuances^15-18^.

There are also a number of studies advocating paid maternity leave and discussing its effect on the initiation and maintenance of breastfeeding^16,19^. However, there is a heterogeneity of reasons for breastfeeding suspension, among them, the most mentioned are: the presence of pain and discomfort when breastfeeding, the lack of practical knowledge to get the baby to latch on, self-reported insufficient milk, the availability of qualified support, bonding to carry out breastfeeding, the training of maternal and child health personnel, the cultural acceptance of breastfeeding, and the development or not of donated human milk banks, among others^20-23^. Apart from the reasons related to maternal health and the return to work, there are few studies that address the processes of breastfeeding from the perspective of mothers.

In this framework, the main objective of this study is to explore and analyze the factors that facilitate or hinder exclusive breastfeeding from the perspective of mothers, considering their socio-educational background and maternity experience.

## METHODS

This study employed a qualitative, exploratory, and descriptive design based on the use of focus group discussions, aimed at understanding the fundamental challenges and strengths of exclusive breastfeeding and its sustainability over time from the perspective of mothers of infants aged 3 to 15 months.

The sample consisted of 32 focus groups. The segmentation criteria were: 1) Maternal experience – primiparous mothers (with a single child aged 3–15 months) and multiparous mothers (with more than one child, and with an infant in that age range at the time of the study); 2) The highest education level achieved – mothers with incomplete secondary education or lower, and mothers with complete secondary education or higher; and 3) Experience of exclusive breastfeeding continuity – exclusive breastfeeding (EBF) until at least the first six months and non-exclusive breastfeeding (Non-EBF) prior to the first six months.

The groups were conducted in different cities in Argentina with the objective of obtaining a sample with a broader range of social segments for analysis. The geographical selection criterion was the EBF level registered in the last National Survey on Breastfeeding^13^. The selected urban areas were Greater Buenos Aires Metropolitan Area (56.3% of EBF); Resistencia, Chaco (65.8% of EBF); San Fernando del Valle de Catamarca, Catamarca (30.4% of EBF), and Gran Córdoba, Córdoba (40% of EBF).

Sample selection and recruitment were conducted by the survey team based on predefined eligibility criteria, including parity (primiparous and multiparous mothers), education level, and EBF continuity.

In each of the four selected Argentine cities – with varying EBF prevalence – key informants from neighborhoods with diverse socioeconomic backgrounds were identified. These informants included community leaders such as coordinators of neighborhood clubs, cultural centers, schools, and religious institutions. They were tasked with identifying and contacting eligible mothers. Snowball sampling was then applied, as initial participants referred additional mothers from their networks.

A total of 32 focus groups were conducted, each including 6 to 10 mothers, resulting in the participation of 234 women. The recruitment and discussion sessions were not held in hospitals or healthcare centers, but rather in neutral, socially oriented spaces – such as coworking venues and communal meeting rooms – specifically arranged to ensure a comfortable, non-clinical environment. This setting was intended to foster openness and trust among participants.

Each session, lasting between 50 and 85 minutes, was facilitated by one of the trained authors using a semi-structured discussion guide. To foster connection among participants, sessions began with short personal introductions (name, age, occupation, and age of the child). Discussions evolved from general reflections on breastfeeding to detailed accounts of factors that facilitated or hindered EBF.

Prior to participation, all mothers provided written informed consent. The sessions were audio-recorded using a mobile app and a voice recorder, with strict anonymity protocols in place. Recordings were then transcribed verbatim for analysis.

Data analysis was conducted through a combination of deductive and inductive coding, grounded in the study objectives and the transcribed content. The initial coding was performed using MAXQDA, followed by further analysis in Atlas.ti. This process resulted in the identification of approximately 79 codes, which were later organized into a set of core analytical categories.

## RESULTS

In the analysis, 31 face-to-face groups were valid (one focus group was conducted virtually), resulting in the inclusion of 231 participants. The participating mothers were aged 16–44 years, with a mean age of 29.5 years (Table 1).

**Table 1:** Demographic and social characteristics of mother participants in the focus groups (N=231)

*Variables*	*Categories*	*Median*
**Age** (years)	Primipara	29.5
	Multipara	32.0
		***n***
**Education level**	Complete secondary education or higher	134
	Incomplete secondary education or lower	97
**Type of maternity**	Primipara	102
	Multipara	129
**Geographical region**	GBA	57
	Córdoba	63
	Catamarca	64
	Chaco	47
**Breastfeeding type**	Exclusive	120
	Non-exclusive	111

The aspects that facilitate and hinder the exclusive breastfeeding (EBF) process are multiple and often interrelated. For the purpose of analysis and presentation, these elements are organized into a set of categories considered relevant to the study (Table 2). Some of these categories were predefined in the guiding instrument used during the focus group discussions, while others emerged inductively from the participants’ accounts. Although facilitators and barriers are not addressed under separate headings, both are clearly identified within each thematic category, allowing for an integrated understanding of the experiences described. The following section provides a category-by-category analysis of these findings (Tables 3 and 4).

**Table 2:** Categories used in the analysis of exclusive breastfeeding (EBF)

*Aspects facilitating EBF*	*Aspects hindering EBF*
Biopsychosocial aspects Characteristics of delivery Family environment Prenatal breastfeeding education and awareness Working conditions	Biopsychosocial aspects Characteristics of delivery and newborn health Family environment Prenatal breastfeeding education Working conditions Sociocultural aspects

**Table 3:** Key quotes from the breastfeeding analysis, organized by analytical category

*Aspects facilitating EBF*
**Biopsychosocial aspect** *‘Truth is he [the baby] needs her breast, her breast milk, and I need his love because it’s the moment in which mother and son bond, and all the problems and what happens around us don’t matter, it’s our moment, our bond, just as I felt it in my belly, it’s very sacred.’* (EBF, primipara, CS, Catamarca) *‘I tried not to get nervous. About breastfeeding, about feeding. The connection. Yes, yes. The connection we have with the breast. Also, one feels relief. Like, for example, I feel a huge relief when the baby latches onto the breast cause usually my breast is very full and gets swollen. So, when I have the baby here it’s like God, this is so beautiful! It’s a beautiful sensation.’* (EBF, primipara, IS, Catamarca) *‘… my partner hints it at me saying that the year she’ll turn two we should leave her at nursery, but no, I’m afraid of leaving her, what’s more, I’m still afraid of leaving her with my mom or my partner sometimes, cause I don’t know what they’ll feed her … I feel like nobody is going to take care of her better than me, that’s how I feel.’* (EBF, primipara, CS, GBA) *‘It’s also an advantage because before when I bottle-fed her, she would cry and cry and I’d try hundred positions to calm her down, my arms would get tired and everything, and when she latched onto the breast, that’s it, she calmed down. So, there’s that advantage, you can calm your baby down without the need of juggling feeding and your safety, just by breastfeeding her.’* (EBF, primipara, CS, GBA)
**Characteristics of delivery and newborn health** *‘I had read about the sacred hour. I had a vaginal birth, as soon as he was out, I started to breastfeed, then they took it away from me to weigh him, then they gave him to my dad and then again to me.’* (EBF, primipara, CS, GBA) *‘Depends on the type of delivery, if it’s a cesarean section it takes longer, but if it’s a normal delivery you can breastfeed the baby right away.’* (EBF, multipara, IS, Catamarca)
**Family environment** *‘And well, he [the baby], for example, when it comes to maternity, what helped me a lot was my mom’s support. She was in the maternity ward, which had only 24 visiting hours, and then after, it was like the next day it was only me alone.’* (EBF, multipara, CS, Córdoba) *‘So, each thing I’d say [to her mother] or question I’d had, I’d ask her, and she would explain it to me.’* (EBF, primipara, IS, Catamarca) *‘… I think that a mother will not like focus only on the baby but also, like, she focuses more on her daughter.’* (EBF, primipara, CS, GBA)
**Prenatal breastfeeding education** *‘There’s nothing like breast milk, antibodies, and all the vitamins, minerals and everything the child needs, the body of the mother prepares itself from the very first minute that pregnancy starts.’* (EBF, primipara, IS, Catamarca) *‘Like, what I am saying is that she [her baby] made me lose weight [she associates it to breastfeeding]. Because I got to lose up to 65 pounds and I still have some pounds left.’* (EBF, primipara, CS, Catamarca) *‘For me the breast is the connection to the heart. And well, also his feeding. It is said it’s different. It’s not the same because it doesn’t have the same vitamins. Everything that one can transmit to the baby through breast milk.’* (Non-EBF, primipara, CS, Córdoba) *‘You breastfeed the baby, and you know it doesn’t hurt the baby [in relation to colics].’* (EBF, multipara, CS, Resistencia) *‘Yes, I went there [to the pre-natal course] since I was 20 weeks pregnant, more or less, before. Four months of pregnancy. And from then on, I went until I had him. There they teach you about … like, it’s two hours long. The first hour is theoretical and informative and the second is practice. They teach you breastfeeding, the positions to breastfeed the baby, they teach you birthing positions, how to breathe, because that helps you a lot when … and a lot of other things.’* (EBF, primipara, IS, Resistencia) *‘And well, both of us [her partner and her] did the courses on first aid, childcare and breastfeeding at the Apapachar space. There we got informed and well, we received our premature baby at six months old. And well, we were already in super parents’ mode waiting for him to arrive and everything related to breastfeeding was beautiful with all the prior education we got to have.’* (EBF, primipara, CS, Catamarca) *‘See, at the clinic where I gave birth I took a course, which starts from the beginning of pregnancy until … the two, five, six, seven months. And it’s like a group of moms. So, I sit and speak. I have moments to clear up my doubts about either breast milk, or medicines or diapers. Anything. I did feel a lot of support from that side.’* (Non-EBF, primipara, CS, Córdoba) *‘I also gave birth at the San Juan de Dios hospital, I also used that system, and even if I didn’t send messages, they would do it asking if I needed something, how I was, just once I went to see her, when the baby wouldn’t latch onto the breast and she helped me, she made me take pictures so that later, when I was alone at home, I could look at the latch, to help.’* (Non-EBF, primipara, CS, GBA)
**Working conditions** *‘Right now, I have the possibility to do telework, which is a lot, because I’m at home and, well, I can take a small break, go to my bedroom, they are taking care of her and, well, I’m there. But when I’ll have to send her to nursery or leave at some point, like, well, I’m getting ready for that moment.’* (EBF, multipara, CS, Córdoba) *‘Right, at least, I don’t know if a leave, because obviously one must go back to work, but fewer hours, so as not to leave him for so long without breastfeeding. They could tell you “Okay, in those 6 hours, we’ll give you one hour so you can leave early”, but in between, they can bring you the baby for a little while. And with that, see, it wouldn’t be so necessary.’* (EBF, multipara, CS, Catamarca) *‘ I try to do things with her [self-employment], like, with pre-sale of milk, diapers, things I see can be profitable, I do them, but everything with her, and I know I have to go back to work, I think maybe when she turns 3 and goes to pre-school, although my partner hints it at me often that when she turns 2 we should leave her at nursery, but no, I’m afraid of leaving her …’* (EBF, primipara, CS, GBA)
***Aspects hindering EBF***
**Biopsychosocial aspect** *‘And since there wasn’t any coming out, I couldn’t get any colostrum coming out and all that. And since I met him [the baby] after the first 12 hours because I had a complicated pregnancy … There wasn’t any milk coming out, so the nurse insisted and when she insisted, he hurt me, he hurt me all over and I cried … he would latch onto only one [breast]. And that’s how it went until later when he didn’t want to anymore. And after 6 months my breast milk dried up.’* (Non-EBF, primipara, IS, Córdoba) *‘Yes, I was a bit scared, because I would say nooo … She [the baby] had a hard time latching on. It was hard for her, I had to put on a nipple shield, and everything because she hurt me, because my nipples hadn’t developed.’* (Non-EBF, primipara, IS, GBA) *‘The same as her happened to me at the start. He [the baby] slept a lot. So, at night I would let him sleep, I didn’t want to bother him. Maybe he slept for 6, 7 hours. And I also had a febricula like two times. Ugh, horrible.’* (Non-EBF, primipara, CS, Córdoba)
**Characteristics of delivery** *‘The first food my son had was formula, because he didn’t latch on, and she [her baby girl] cried with hunger, so she was given 5 ml of formula, and then I didn’t want them to give her more formula, I wanted her to be breastfed, we tried for a week and it wasn’t possible, I would also express my milk and bottle-feed her, but it wasn’t the same and she would gain very little weight, my milk came in late, they told me it was because of the cesarean section, I never felt the milk strongly coming in, after the first week of birth my daughter cried with hunger so I decided to feed her formula.’* (Non-EBF, primipara, CS, GBA) *‘They didn’t give me the baby because I was weak, I couldn’t hold him because they said he could fall. But they did come in and they placed him on my breast because he has to feel the warmth of his mother.’* (Non-EBF, primipara, IS, Córdoba) *‘In my case I had a cesarean section, I had a checkup and was told I had to have it, it was a bit chaotic and as soon as he was born, they latched him onto my breast, they asked me which breast I wanted to feed him from. I would have felt very sad if I couldn’t have had that first latch.’* (Non-EBF, primipara, CS, GBA) *‘My baby spent hours in Neo, because when he was born his heart was beating a little faster, he was in Neo for a couple of hours and there he was given formula, well, I had a cesarean section too, I couldn’t stand up yet and I had to wait to be discharged so I could get up and go upstairs. During the first hours there, he was fed, like, a booster. And only when I was able to get up could I breastfeed him.’* (EBF, primipara, CS, GBA) *‘Were you given the baby to breastfeed? If it’s a natural birth, yes, if it’s a C-section, they don’t leave you until the next day.’* (Non-EBF, multipara, IS, GBA) *‘So, passed …, I was in ICU for a week and a half. So, I didn’t breastfeed her or anything else. I felt like my breasts were exploding. Sometimes I would get lumps. So, I would massage them. A lot of things. Express and freeze milk. Those kinds of things. And … and well, and Julieta liked formulas. Until she finally latched onto the breast, I suffered a hell of a lot. A hell of a lot.’* (EBF, primipara, CS, Córdoba) *‘Because as soon as I left the OR, I couldn’t breastfeed. I couldn’t do anything. So, they bottle-feed her right away.’* (EBF, primipara, CS, Catamarca)
**Family environment** *‘I breastfed my first daughter and had like 10 people sitting around me looking at the situation. Yes. No, it stresses you out a lot. My sister, my mom, they were all there keeping an eye on me. Sure, she was the first. They were keeping an eye on her. It’s okay, they have good intentions, right? Yes, yes. Everybody acts out of love, but sometimes they don’t. No. With the first one I felt much more pressured.’* (EBF, multipara, IS, Córdoba) *‘My mom, my aunt, my grandmother, they inform but also misinform and cause fear and insecurity also; “yes, don’t give the baby that, you don’t feed yourself, feed the baby formula”.’* (EBF, primipara, CS, Catamarca) *‘[her mother] she tells me, “there can be an emergency or something, or you have to do something and I could take care of her”. She says “but since you exclusively breastfeed her, it’s up to you. Wherever you go, you must take her too. She has an emergency or something and you have to take her”.’* (EBF, multipara, IS, GBA) *‘I took care of her all morning from when my husband was working until he came back. And it was like, well, at night, I mean, like, have a shower, dress up, get comfortable, and well, I want to sleep a couple of hours so I wouldn’t breastfeed the baby, he [my husband] got the formulas ready and fed the baby at night. So, he would take care of the baby at night, and later in the morning, I would do it again.’* (Non-EBF, primipara, CS, Córdoba) *‘And he would say to me, yes, but … “I want to sleep a little bit because I must go to work in a few hours” [her husband would say that to her]. And I say, “but no”. I say, “I can’t do it, I mean, help me”. I mean, What do I do? So, I went into a sort of crisis. I didn’t know what to do. I mean… you don’t know what… I mean, the two of us. We didn’t know how to. We didn’t know how to. Like, I would get sad. I fought with him.’* (Non-EBF, primipara, CS, Córdoba)
**Prenatal breastfeeding education** *‘In my case, I didn’t have to consult anything, my idea has always been to breastfeed, my milk came in early, so I was already exploding. From videos, I would see a lot on TikTok, but I wasn’t interested in doing pre-natal courses and all that, because my mindset was “whatever happens, happens”.’* (Non-EBF, primipara, CS, GBA)
**Working conditions** *‘At first there was consideration, in reality the first week I said to them “he is not going many hours”, it’s 4 hours at nursery, and I have to pick him up at midday, I work 9 hours, so they told me there was no problem, that when I needed to go pick him up, I could go. That was the first week, when I went to pick him up, I kept on working from home, and later the next week they told me, “well, I guess he has already got adapted, so today you work full time”; in reality, he doesn’t easily get adapted in one week, plus he only goes twice a week, so there’s consideration, anyway, now he has bronchiolitis, and with the certificate they don’t ask me to work onsite, but like there is consideration, but also they require you to be there more.’* (Non-EBF, primipara, CS, GBA) *‘See, just yesterday I went to work to look for the papers. I work 12 hours, so I asked, “I mean, now that I have a baby, apart from the breastfeeding break and everything, will I be able to change roles?”. “No, you left doing a shift and you come back on the same shift”. That’s why it’s gonna be very hard for me, it’s 12 hours, like, I’ll leave the baby the entire day. I would love not having to go to work, but what happens is that I earn more than my husband, the rent. No, but also … but it’s also social pressure, right?’* (EBF, multipara, CS, Córdoba) *‘I started to work after the first and a half month with a lot of crises, new job, new course of studies and everything, many new things, a lot of information and it was pretty hard for me to comply with the 6 months. Every month I would be like “well that’s it, feed her formula”, and it was like that until the 6 months, 7 months, when I said “that’s it” … This feeling of, like, I really needed to, at least, sleep first, I wasn’t sleeping at night, go to work and at least not having to feel uncomfortable at a new job, having to ask for permission … and I couldn’t feed her enough, I felt very very pressured, it took that pressure off me, now she drinks formula and that’s it, but well. My heart aches, I would’ve loved to continue breastfeeding.’* (EBF, primipara, CS, Córdoba) *‘I woke up at 6 in the morning, went to the workplace, the place wasn’t a bit practical or comfortable for me to express milk. So, when I had to express milk, the more nervous you get, the less milk comes out, the less … Even if the breast pump is electric or made by NASA, it doesn’t come out as it would if the baby was breastfed. So, I’d go home from work after seven hours with this [“little bit” hand gesture] and it was frustrating because the following day I had to feed that to my baby and it wasn’t enough, so, since then I started thinking … “What are we going to do?” And the baby isn’t eating … It’s a real concern …’* (EBF, primipara, CS, Córdoba) *‘Working and not having a place to express milk ruined breastfeeding for me.’* (EBF, primipara, CS, Córdoba) *‘Well, I don’t know, maybe if I hadn’t worked, I would have exclusively breastfed him, I would’ve gotten him used to me. But well, life is not perfect during this time.’* (Non-EBF, primipara, IS, Córdoba)
**Sociocultural aspects** *‘[She was afraid] of her not getting full of my milk, cause, how can I say this, I listened to all the advice and there were people saying, “but you are so skinny, your baby won’t be full, he will cry all day”. And like I would overthink those comments a lot and I was afraid of not getting my baby full.’* (EBF, primipara, IS, Resistencia) *‘… what they [family] tell you is “well, you have to breastfeed for 6 months, then there’s no milk”. “No, you are breastfeeding him, you have to stop because that is water now, it doesn’t feed him, you are hurting him”, and it becomes a vice.’* (EBF, multipara, CS, Catamarca) *‘So, like, look, that baby is hungry, you are not breastfeeding him. Look, you must attach him to your breast all the time.’* (Non-EBF, primipara, CS, Córdoba) *‘It happened to me at bars; one time a woman came in and said, “you can’t do that in here” and I said to her “What can’t I do?” .’* (EBF, primipara, CS, Catamarca) *‘I wasn’t embarrassed to do it and there was one guy looking at me like this … and that’s when I started to cover myself.’* (Non-EBF, primipara, IS, Córdoba) *‘Me too, not because I was particularly ashamed of it, but I feel very embarrassed about breastfeeding anywhere where my family is not, and I saw very few public spaces with lactation rooms, only at the Once railway station. At work, I pump my milk in the disabled bathroom, but there is not a special place or where to store it either, only the refrigerator of the whole office.’* (Non-EBF, primipara, CS, GBA)

EBF: exclusive breastfeeding. CS: complete secondary education or higher. IS: incomplete secondary education or lower. Source: Authors’ own elaboration based on 32 focus groups conducted in 2023.

**Table 4:** Summary of main findings according to analytical categories

*Aspects facilitating EBF*	*Aspects hindering EBF*
**Biopsychosocial aspects** Patience during the initial process. Being at peace during breastfeeding moments. Enjoying the attachment bond with the baby. **Characteristics of delivery** Having a vaginal delivery. Respecting the first hour of mother-infant contact. **Family environment** Post-partum support: a supportive family (‘being capable of asking for help’). **Prenatal breastfeeding education** Prior decision of the mother and her environment. Intention to develop self-confidence and breastfeeding skills (developing nipples prior to childbirth; finding a position to hold the baby; participating in educational facilities and socializing with peer groups). Maintenance of the decision to breastfeed (EBF benefits: immunity, nutrition, digestion, bond, healing power). Value practicality (no washing, no using water, no heating milk, no leaving home with extra elements, etc.), and the economic benefit. Learning to express and handle milk. Learning to build a breast milk bank. Access to prenatal breastfeeding education with trained professionals (obstetricians, midwives, childcare specialists, liaison nurses and pediatricians). Free childcare services. Open consultation spaces with health professionals (WhatsApp chat). Access to peer group activities to share values and concrete experiences. Access to content from pediatricians or other professionals on social media (TikTok). **Working conditions** Leave extensions. Working from home. Reduction of working hours. Lactation breaks. Lactation rooms at work and public spaces.	**Biopsychosocial aspects** Mother’s persistent physical pain (cracks, mastitis, milk blab). Post-partum depression. Physical and psychological exhaustion during the first month (guilt, sadness, frustration, failure, confusion, anxiety). Perception of insufficient milk (not enough quantity, no food value in breast milk). Isolation (being unable to ask for help and receive help, no socializing with peers, no consulting others). **Characteristics of delivery** Having a cesarean section. Requiring neonatal intensive care. Newborn weight loss during the first weeks of birth. Behavioral aspects of the baby (long sleep, no latch, rejection). **Family environment** An invasive family environment which judges the mother. ‘Weak’ ties for support purposes (no helpful and supportive environment). Mother’s loss of autonomy (being unable to leave the baby with other people). Insufficiency or lack of paternal role in facilitating breastfeeding. No care and domestic help from other members of the household. **Prenatal breastfeeding education** Poor milk handling and storage practices. Limited access to health services, knowledge and education. **Working conditions** Working in the informal market economy (no rights). No flexible work. **Sociocultural aspects** Social idealization of breastfeeding. Myths about breast milk. Stigmatization of breastfeeding in public spaces.

EBF: exclusive breastfeeding. Source: Authors’ elaboration based on 32 focus groups conducted in 2023.

### Biopsychosocial aspects

*Barriers*

Participant mothers frequently identified biopsychosocial factors that hinder the maintenance of EBF. These include physical discomforts, such as pain, dermatitis, and nipple damage, which often lead to early abandonment of breastfeeding. Additionally, many mothers reported experiencing postpartum depression, psychological distress, and a sense of isolation, especially during the first month after birth. Feelings of insecurity when caring for the baby also emerged as common obstacles, particularly among first-time mothers. These challenges were often exacerbated by the lack of practical knowledge and emotional support, making it harder to persist with breastfeeding.

*Facilitators*

Despite the difficulties, mothers also described certain internal resources and attitudes that helped them maintain EBF. Several participants emphasized that breastfeeding is a demanding process that requires patience, calmness, and the ability to enjoy the bond with the baby. Recognizing breastfeeding as a learned skill – rather than something automatic – empowered some women to seek knowledge and support, which in turn fostered greater confidence and perseverance in continuing EBF.

### Characteristics of delivery and newborn health

*Facilitators*

Delivery conditions were identified by mothers as an important factor influencing the initiation of breastfeeding. According to their accounts, vaginal delivery often facilitated the early latching of the infant onto the breast, especially when the first hour of mother–infant contact was respected. Mothers emphasized that immediate contact with the baby contributed significantly to the successful initiation of breastfeeding.

*Barriers*

In contrast, mothers who underwent cesarean sections commonly reported that their babies were initially fed with formula milk and experienced difficulties latching onto the breast. This was attributed to delayed physical contact due to the medical attention the mothers required post-surgery.

Additional barriers arose when the newborn required neonatal intensive care, which similarly delayed mother–infant contact and led to early formula feeding. Comparable challenges were mentioned when the baby was underweight or exhibited behaviors such as excessive sleepiness, refusal to latch, or breast rejection during the first hours of life.

From the mothers’ perspective, these factors often interacted with the lack of immediate contact, resulting in the early introduction of alternative milk. Furthermore, infant weight loss during the first days after birth was considered a relevant issue, frequently leading to ‘mixed feedings’ – the combination of formula and breast milk – as a perceived solution.

### Family environment

*Barriers*

In primipara mothers and in households with a low level of education, lack of family support, or the presence of ‘weak’ support ties (i.e. the absence of a helpful and supportive environment), combined with physical problems and psychological distress, can negatively affect the maintenance of EBF. Mothers also expressed that, due to their insecurity in caring for the baby and the challenges in feeding and satisfying the baby’s appetite, the intervention of family members – typically other women such as their own mothers or mothers-in-law – was often invasive and counterproductive. These interventions sometimes generated psychological distress, feelings of guilt, and a sense of failure, hindering the process of learning to breastfeed.

Some mothers reported that in such environments, decisions about infant care were made by others, reducing the mother’s autonomy. This was particularly noted among mothers with a low level of education, but also appeared among multipara mothers who were burdened with additional care and domestic responsibilities, and therefore relied heavily on the help of other women in the household.

*Facilitators*

In these contexts, having learned how to express and safely store breast milk was considered an advantage, as it allowed other household members to participate in the care of the baby, and the baby could still be fed with breast milk.

### Prenatal breastfeeding education

*Facilitators*

The information women could obtain before labor became pivotal for the development of self-confidence and the skills necessary for maintaining breastfeeding. Expectant mothers who participated in maternity support programs reported acquiring valuable competencies, such as nipple preparation prior to labor, finding suitable breastfeeding positions, expressing and handling milk, and even building a breast milk bank. In addition, the knowledge of breastfeeding benefits for both baby and mother was a source of motivation. Although most participants had access to group activities, longer duration programs were considered especially influential.

Support during the post-partum period also played a critical role, particularly for primipara mothers, who described this time as full of anxiety, fear, and uncertainty. In this context, the presence of trained professionals (e.g. obstetricians, midwives, childcare specialists, liaison nurses, pediatricians) and access to maternity care structures facilitated EBF initiation and continuation. Open consultation spaces, WhatsApp chats, and postpartum activities offered mothers real-time guidance and emotional support.

Additionally, all mothers – both those who continued and those who discontinued EBF – mentioned that its practicality was a strong motivator. The absence of the need to heat water, sterilize bottles, or pack extra items when leaving the house simplified daily life, especially in households with more than one child.

A widely used and valued source of information was social media, particularly TikTok, where mothers accessed content from pediatricians, professionals, and peers, reinforcing and legitimizing breastfeeding practices.

*Barriers*

However, some participants described significant barriers. Limited access to health services was reported as a key factor that restricted their ability to gain sufficient knowledge and education to build confidence and sustain breastfeeding. This lack of access also made it harder for some mothers to legitimize EBF in family or social settings.

Another major barrier mentioned was physical pain and discomfort, especially at the beginning of the breastfeeding process. Many mothers stated that pain, in combination with insufficient information, left them without the tools or strategies to manage difficulties – ultimately leading to the early cessation of EBF.

### Working conditions

*Facilitators*

Returning to paid work is one of the most compelling explanations for not continuing with EBF. However, mothers who had access to maternity leave and flexible work arrangements were generally able and willing to continue breastfeeding. Among mothers with a higher level of education, various strategies were employed to sustain EBF, including the use of paid or unpaid maternity leave, remote work, and reduced working hours. These conditions functioned as facilitating factors, enabling the continuation of breastfeeding alongside work responsibilities.

*Barriers*

Conversely, mothers with precarious jobs, where lactation rights were not respected or where minimum conditions were not met, reported feeling forced to abandon EBF. This was particularly true for women in the informal labor market or those without adequate job protections.

Mothers with a low level of education who worked before pregnancy, often chose to leave the workforce to focus on caring for their child, while those in more formal settings – especially with higher level of education – faced a dilemma upon returning to work.

There was widespread agreement on the difficulty of combining EBF with paid employment. Reported tensions included: delegation of feeding decisions to other caregivers, challenges with milk expression and storage, psychological distress from negotiating breastfeeding rights at work or within the family, and personal fatigue or unmet needs.

### Sociocultural aspects

*Barriers*

In certain contexts, particularly among primipara mothers, participants reported experiencing social pressure rooted in the notion that breastfeeding is directly linked to being a ‘good mother’. This perception created emotional stress and heightened expectations around the breastfeeding experience.

In low education environments, persistent myths such as the belief that ‘breastfeeding does not feed the baby’, undermined mothers’ confidence in EBF and often led to encouragement to introduce other milk as a supplement.

Additionally, in some provincial cities, mothers reported stigmatization of breastfeeding in public spaces. While not a formal or structural barrier, this was widely described as an uncomfortable and inhibiting experience, which discouraged breastfeeding in public and contributed to feelings of exposure or shame.

*Facilitators*

No significant facilitators were reported in this category. However, the discomfort expressed by mothers highlights an opportunity for cultural and policy interventions aimed at normalizing breastfeeding in public and challenging harmful myths.

Based on the interviews, it is observed that there are concrete processes in the experiences of mothers that facilitate or hinder EBF initiation and, primarily, its maintenance. A summary of the results, which were systematized based on the central categories surrounding both EBF processes, is given in Table 4.

## DISCUSSION

The main research findings are in line with previous BF research showing that what mostly conditions BF initiation and maintenance are not the biological and health-related aspects, but rather the cultural aspects, since BF not only depends on working conditions, but also, primarily, on BF public policies, the availability of support groups, social media use, and lobby groups^1,22^.

It is known and as this study confirmed, the prior decision made by the mother and her environment is fundamental for the initiation and continuity of exclusive breastfeeding (EBF) over time, but this decision is made before the baby is born^2,24^. Family and professional support also play a key role in the initiation of breastfeeding and in providing support for its continuation, issues that were also evidenced in the present study^4,25^. In the case of Argentina, this is further reinforced by the importance of peer group support and exchange.

Although family environment, both the role of the father and close family, offers opportunities for the mother to have post-partum peace and enjoy maternity^3,24,26^, in diverse cases, as shown by the present study, this environment can turn into a barrier, especially when it is invasive or the support ties are weak. The research data show that the family environment, especially that of the maternal grandmother and/or the primipara mother’s sister, focuses on the mother’s loss of autonomy, in the face of her inability to leave the baby in the care of others. This becomes a process hindering EBF, as it influences the introduction of formula milk as the concrete solution to the need of leaving the baby at the care of others, turning family into a lobby group^24,27,28^.

Moreover, as shown by international research, women expect breastfeeding to happen ‘naturally’ and easily, but they reported that once they were faced with the situation, they did not feel prepared for the challenges BF presented^28^. Those who attended classes and courses were provided with more tools to overcome the physical pain associated with the initiation of breastfeeding and the characteristics of infant feeding, which is consistent with the findings of other studies^21-23^. As a finding, it can be claimed that those who attended long-term courses and had professional post-partum support were provided with tools that enabled them to meet these needs and overcome the first instances that usually hinder BF.

Returning to paid work is one of the key factors for EBF cessation^10,24^. This study reveals a paradox: while informal and precarious work is generally associated with vulnerability and limited rights, in the context of early motherhood, it may provide certain conditions such as flexibility and the ability to pause employment that unintentionally support the continuation of exclusive breastfeeding. For some women, especially primiparas, the low opportunity cost of leaving poorly paid informal jobs makes continued breastfeeding a more viable and even preferable option.

Another key finding of this study is that EBF maintenance is different for multipara mothers and primipara mothers. As multipara mothers have other children to take care of, the domestic workload is multiplied, and because they have previous experience, they present greater flexibility in their decision-making regarding EBF. For multipara mothers, formula milk opens the possibility of having time and leaving children in the care of others, and their greater individual experience shows them that children grow up, either way, healthy. Hence, it was observed that one of the differences in EBF maintenance or not, among primipara and multipara mothers, is not evoked by having previous breastfeeding experience, but rather by watching children grow.

As shown by the latest research, it was also observed that social media is a space not only for socializing with other mothers sharing the same experience, but also for searching for updated information^3,6,29,30^. Social media, primarily TikTok and Instagram, offer the opportunity for breastfeeding support from peers, mother-to-mother, including the incorporation of informal interactions which create ‘momentary ties’ of support. By offering the opportunity for immediacy and anonymity, social media allow greater communication about personal choices related to infant feeding methods and breastfeeding complications, as well as the sharing of values and concrete experiences, and a space to ask questions to peer groups^5,30-32^. Although in this study, as in others, primipara mothers are the ones who use social media the most^33^, it is important to stress that searching for information prior to infant birth is valued by all mothers as a process facilitating breastfeeding. The present study shows that multipara mothers, though experienced, also seek updated information, since their children were born years ago, and this new information entails a different view of breastfeeding, and also, because of the difference in ‘how babies are nowadays’ compared to before. Mothers’ comprehension of maternity norms is shaped by digital interactions. Therefore, online relationships are a key source of support for all mothers. As it was observed, information and support are two key aspects of breastfeeding; hence, social media spaces become a great option to promote breastfeeding through information sharing and the creation of positive social connections. Social media can be effective in providing a type of support that counteracts the processes hindering EBF and can also be incorporated into family groups.

The mothers’ assessment of the practicality of EBF is also deemed a finding of this study. The totality of mothers, both those who exclusively breastfeed and those who do not, emphasizes the practical advantages of BF for everyday life (especially during the first months and at night), since it is not necessary to consider or organize extra equipment to feed the baby. This aspect can be a great advantage for future work on actions to promote EBF.

### Limitations

Among the main limitations of the study are its qualitative design and focus on urban areas, which restrict the generalizability of the findings to other populations, particularly those in rural settings. Additionally, the study relies solely on the perspectives of mothers, without incorporating those of healthcare professionals, employers, or other key stakeholders. There may also be participation bias, as the women who agreed to take part in the focus groups might have been more engaged or sensitized to the issue of breastfeeding.

## CONCLUSIONS

This study highlights that exclusive breastfeeding (EBF) is facilitated by access to information through participatory formats such as interactions with health professionals and peer support groups, which empower mothers in both social and work environments. In contrast, barriers such as insufficient postnatal support, physical and emotional discomfort, family pressures, and work-related challenges hinder EBF continuation. The findings underscore the need to raise social awareness and promote shared responsibility among families, employers, and society.

## Data Availability

The data supporting this research are available from the authors on reasonable request.
